# Comparison of Health Outcomes by Care Provider Type for Newly Diagnosed Mild Type 2 Diabetes Patients in South Korea: A Retrospective Cohort Study

**DOI:** 10.3390/healthcare10020334

**Published:** 2022-02-09

**Authors:** Hee-Chung Kang, Jae-Seok Hong

**Affiliations:** 1 Department of Health Care Policy Research, Korea Institute for Health and Social Affairs (KIHASA), Sejong City 30147, Korea; hee-chungkang@kihasa.re.kr; 2 Division of Health Administration and Healthcare, Cheongju University College of Health and Medical Sciences, Cheongju 28503, Korea

**Keywords:** primary care, type 2 diabetes, quality of care, usual source of care

## Abstract

(1) Background: Although the beneficial impacts of primary care on patients’ health outcomes have been reported, it is still difficult to limit patients’ herd behavior in seeking tertiary or large hospital services in South Korea. The purpose of this study was to examine whether the use of primary care clinics was longitudinally beneficial for mild diabetes. (2) Methods: Using claims data from the National Health Insurance (NHI) program, this population-based retrospective cohort study compared health outcomes over a 4-year period from 2011 to 2015 by type of health care institution as a usual source of care in diabetic patients who were newly diagnosed in 2011, i.e., without any diagnosis between 2005 and 2010. (3) Results: Compared to those attending hospitals, general hospitals (GHs), or tertiary general hospitals (TGHs), patients who visited primary clinics were more likely to experience better health outcomes such as the lower risk of hospitalization and death. (4) Conclusions: These results provide additional evidence that higher-value from primary care clinics would be highly expected for early and mild type 2 diabetics. Promoting the Informed, patient-centered decision toward primary care clinics would contribute to improving the value of the healthcare delivery system.

## 1. Introduction

The number of patients diagnosed with diabetes has soared worldwide with strong economic growth and an improved standard of living, and the socioeconomic cost of diabetes is also increasing rapidly [1]. The global prevalence of adult diabetes has nearly doubled from 4.7% in 1980 to 9.3% (approximately 463 million) in 2019, caused 4.2 million deaths in 2019, and it is projected that, by 2045, up to 700 million adults will have the condition [1,2,3,4]. South Korea is not an exception because 13.8% of Korean adults aged ≥30 years had diabetes in 2018 [3]. The World Health Organization (WHO) has thus designated diabetes as “one of four priority noncommunicable diseases (NCDs) targeted by world leaders” in the 2011 Political Declaration on the Prevention and Control of NCDs [1]. The United States, British, and Australian governments and others have introduced active management programs centered on primary care, provisions for quality services, continuous management, and improved health outcomes for diabetic patients based on the latest scientific findings [5].

Diabetes is a typical ambulatory-care-sensitive condition for which timely and effective outpatient management can help to avoid complications and exacerbations and prevent hospitalization [6]. Primary care is of core importance for efficient diabetes management [7,8].

Over the past decade in Korea, the government has attempted to transition toward an integrated care delivery system centered on primary care clinics. However, patients’ preference for hospital outpatient departments over primary care clinics has continued to increase, and thus this remains a challenge for policymakers. It is because all patients, under the universal coverage of the National Health Insurance (NHI) program, could choose healthcare institutions which they want to be treated for their health conditions [9]. According to number of beds, the Medical Service Act classifies healthcare institutions into clinics (fewer than 30 beds, main objects which the government has encouraged to play a central role to enhance primary care), hospitals (30–99 beds), and general hospitals (GHs) (more than 99 beds) [5]. The NHI program has two phases of the healthcare delivery system [5]. Only those tertiary general hospitals (TGHs) which are designated by the Ministry of Health and Welfare among the general hospitals qualifying for teaching hospitals and other requirements are permitted for the second phase of care, and the other institutions would compete for the first phase of care [5]. However, outpatient hospital care in Korea has been on the rise for the last few decades. This is related to the overall expansion of the hospital sector [4]. While the outpatient expenditure of clinics increased by 80% between 2005 and 2015, the expenditure for outpatient hospital care has increased by 173%. Consequently, avoidable admissions for diabetes reported 224 as age-sex standardized rates per 100,000 population, nearly twice the OECD average of 127 [4].

The five diseases for which Koreans most frequently seek care at larger hospitals include three conditions: the common cold, diabetes, and hypertension [10,11]. Patients who can be adequately treated at primary care clinics were assessed to make up 32.5% of all outpatient patients who visited TGHs in Korea, indicating the concentration of patients with mild conditions in tertiary care [12], but in a recent study, reportedly, the proportion decreased to 15% [9].

The high numbers of mildly ill patients seen at large hospitals necessarily compromises the efficiency of medical resource allocation, and the patients themselves do not stand to benefit much in terms of outcomes due to the long wait times and short consultations with doctors. Most critically, this phenomenon compromises primary care, increases unnecessary use of hospital outpatient services and wasteful spending within the health care system, and decreases the value of care [11,12,13,14]. These findings suggest that the public should be better informed that primary care clinics can achieve superior outcomes and provide more value to low-risk chronic patients.

This study investigated whether, as a usual source of care (USC), primary care clinics provide better outcomes for mild patients with newly diagnosed type 2 diabetes compared to hospitals, GHs, and TGHs.

### Literature Review

Most previous studies have reported that it is possible to prevent and minimize type 2 diabetes complications or hospitalization if it is treated appropriately in primary care over time based on benefits of continuity of care [15,16]. An increase in diabetes prevalence has been most concerning because it would increase the burden of chronic and acute disease in the general population, with increased demand on health services and economic costs [2,16]. On that note, longitudinal outcomes measurement such as the risk of hospitalization, complication, and all-cause or cause-specific death among defined patient cohorts with diabetes have been recognized as an important tool to improve the quality of primary diabetes care [16]. A relevant systematic review reported that the current understanding of the international burden of and variation in diabetes-related complications was poor, but the available data suggest that rates of myocardial infarction, stroke, and amputation are decreasing among people with diabetes, in parallel with declining mortality [16]. Meanwhile, a recent study suggested a need for more focus on the healthcare journey of people with diabetes and to validate that those best practices in the primary care network structure would optimize healthcare costs while not compromising health outcomes for them on the value-based perspective [17]. Although the beneficial impacts of primary care on patients’ health outcomes have been reported, it is still difficult to limit patients’ herd behavior in seeking tertiary or large hospital services in South Korea. In relation to the informed choice of people with diabetes for right-siting, the more direct comparison on outcome quality of diabetic care between primary care clinics and hospitals in the NHI delivery system has been needed. However, except for studies comparing the quality of care between general practitioner and diabetes specialists on a same primary care level [18,19], it was difficult to find studies relevant to comparing longitudinal outcomes between primary care clinics and hospitals. It is presumably because there have been concerns whether insufficient risk adjustment for patients of hospitals who might have higher risks than those of primary care clinics would result in biased research or not.

There were two relevant studies recently in Korea. One was about the investigation on the primary care patients’ preference for hospitals over clinics, reporting that approximately 15% of outpatient visits of the patients who were eligible for primary care in Korea happened in hospitals [9]. The other compared the overall status of diabetes control and screening for diabetic microvascular complications in patients with type 2 diabetes mellitus between primary care clinics and tertiary general hospitals, reporting suboptimal management in clinics [20]. However, the former did not compare health outcomes of care for patients with diabetes, and the latter was performed only for the small sample of two tertiary general hospitals cross-sectionally. Therefore, it is difficult to compare the results with this study. the further research will have to be performed in the future.

## 2. Materials and Methods

### 2.1. Study Design and Data Source

We used the claims and qualification data of the National Health Insurance (NHI) program, which cover the entire Korean population, for the period of 2005 through 2015. We performed a population-based retrospective cohort analysis on the 4 years of care provided for adult patients aged 20 years or older who were newly diagnosed with type 2 diabetes (E11) in 2011. Newly diagnosed diabetes refers to type 2 diabetic patients who did not have records of being diagnosed with diabetes from the year 2005 through 2010.

### 2.2. Study Population

There were 361,617 patients aged 20 years or older who were first diagnosed with type 2 diabetes (E11) in 2011 in Korea. Of these, 281,077 patients were excluded from our analyses for the following reasons. First, the 169,361 patients who had fewer than four outpatient visits during 3 years after their first diagnosis were excluded, which was to ensure the validity of diagnosis from the NHI administrative claims data [5]. Second, the 15,975 patients who had ever been hospitalized for ischemic heart disease, stroke, renal disease, or cancer during the 12 months before their first diagnosis were excluded. It was to prevent the effects that those would increase patients’ risk and thus might influence his/her choice of healthcare institutions [21]. Third, the 2970 patients who died in less than 3 years following diabetes diagnosis were excluded. Fourth, the 92,771 who did not visit the same health care institution during the 3 years after their first diagnosis were excluded. It was to purify the effects of the USC type on patient outcomes. Finally, study subjects were defined as the patients who survived and continued to use the same health care institutions in the 3 years following their diagnosis (*N* = 80,540) were grouped according to their USC type.

### 2.3. Study Variables

The main independent variable was the type of USC frequented by patients for diabetic care in the 3 years following their diagnosis. The five types of USCs included as variables were TGHs, GHs, hospitals, clinics, and public health centers (PHCs). Healthcare institutions are classified by the Korea National Health Insurance according to number of beds as clinics (fewer than 30 beds), hospitals (30–99 beds), and general hospitals (GHs) (more than 99 beds). Tertiary general hospitals (TGHs) are general hospitals that the Ministry of Health and Welfare designates when they meet standards of teaching hospitals and other requirements [5].

To understand outcome variances, the average number of outpatient visits and the level of medication adherence in the 3 years of diabetic care following diagnosis were calculated according to USC type, as these are typical measures that reflect treatment/care quality. Medication adherence was measured in terms of the medication possession ratio (MPR), which ranged from 0 to 1 [22]; the closer it is to 1, the better the patient’s adherence to his/her medication regimen. The MPR score for diabetes was expressed as the proportion of aggregate days a patient had access to diabetes medications during the study period (MPR = number of days in the study period covered by the supply of medication/number of days in the study period) [22].

The outcome variables were diabetes-related hospitalization and all-cause death observed during the span of time subject to our analyses. In particular, we differentiated hospitalization according to the number of years since diagnosis (1–4 years). Diabetes-related hospitalization included hospitalizations caused by cardiovascular disease (CVD) and renal disease, as typical comorbidities of diabetic patients, as well as diabetes itself. We measured mortality as that observed during the fourth and final year of our analyses, after 3 years of presumably continuous diabetic care.

Confounding factors included the patient’s sex (male or female), age (20–44, 45–54, 55–64, or 65+ years old), residence (Seoul, other metropolitan city, or city/county), the Charlson Comorbidity Index (CCI, 0, 1, or 2+) for the year preceding the initial diagnosis, and income level (Medical Aid, Decile 1-2, Decile 3-4, Decile 5-6, Decile 7-8, Decile 9-10). The CCI was measured based on all diagnostic information for 1 year before the first diagnosis with type 2 diabetes in 2011. The income distributions of patients insured by the NHI were divided into equal deciles (1–10), with the highest decile associated with the highest income level, apart from medical aid beneficiaries.

### 2.4. Statistical Analyses

We performed a chi-square (χ^2^) test and analysis of variance to examine whether diabetic patients’ choices of USC varied with patient characteristics and if their outpatient utilization varied with USC type. We compared outcomes by USC (i.e., institution type) through multivariate logistic regression analyses controlling for patient characteristics. The odds ratio (OR) of hospitalization 1, 2, 3, and 4 years after the initial diagnosis and the all-cause death at 4 years were estimated for the study subjects (*N* = 80,540). In addition, logistic regression was applied to the modified subject group (*N* = 79,000), which comprised only mild patients; those with hospitalizations (*N* = 1540) within 1 year following their diagnosis were excluded. Within each patient group, we compared the patients with a CCI of 0 against all other patients. Statistical analyses were conducted using SAS statistical software (version 9.3 for Windows; SAS Institute, Cary, NC, USA).

## 3. Results

### 3.1. Patient Characteristics by USC Type

For patients newly diagnosed with type 2 diabetes, clinics were the most favored (65.7%) as a USC, followed by GHs (14.9%), TGHs (9.1%), hospitals (8.0%), and PHCs (2.4%) (Table 1). The proportion of female patients was higher in the clinic and PHC categories. The mean age of patients was lowest for those who sought treatment in TGHs (mean age, 53.2 years) and was highest in PHC goers (mean age, 62.3 years). The percentages of patients under the age of 45 were relatively high for TGHs, GHs, and hospitals, whereas clinics and PHCs had more patients aged 65 years or older. TGHs also had a lower percentage of patients on medical aid and a higher percentage of patients in the highest income deciles (9 and 10) than other types of USCs. Patients using TGHs as their USCs were more likely to be living in Seoul, the capital city, and to have a CCI of 2+, compared to patients using other USC facilities.

### 3.2. Outpatient Care Use by USC Type

Table 1 shows that the frequency of outpatient visits and the level of medication adherence differed by USC type. Clinic goers (mean ± standard deviation [SD], 23.2 ± 19.4) had a higher number of outpatient visits on average than those of hospital (18.0 ± 15.5), GH (15.4 ± 13.1), and TGH (12.1 ± 10.6) goers. The MPR was also highest among clinic goers (mean ± SD, 0.42 ± 0.42) than those for hospital (0.37 ± 0.39), GH (0.41 ± 0.40) and TGH (0.34 ± 0.40) goers.

### 3.3. Health Outcomes by USC Type

The ORs of diabetes-related hospitalization 1 year following the new diagnosis were 5.33 (95% confidence interval (CI), 4.72–6.03) for GHs, 4.54 (95% CI, 3.90–5.28) for TGHs, and 2.68 (95% CI, 2.24–3.21) for hospitals, significantly higher than that for clinics (Table 2). As the observation period was extended to 4 years, the ORs scaled down to the point where some changes became nonsignificant. The ORs of hospitalization within 3 years following diagnosis were 3.53 (95% CI, 3.24–3.84) for GHs, 3.03 (95% CI, 2.72–3.37) for TGHs, and 2.09 (95% CI, 1.84–2.36) for hospitals, significantly higher than that for clinics. The ORs at 4 years remained significant only for GHs and TGHs, specifically, 2.85 (95% CI, 2.64–3.10) and 2.44 (95% CI, 2.21–2.68) higher than that for clinics, respectively.

In patients with a CCI of 0, the ORs of hospitalization in hospital goers were similar to the foregoing results; however, those for TGHs and GHs increased. The ORs of hospitalization within 1 year following diagnosis were 6.90 (95% CI, 5.67–8.40) for GHs, 6.09 (95% CI, 4.77–7.77) for TGHs, and 2.63 (95% CI, 1.96–3.54) for hospitals, significantly higher than that for clinics. As the observation period was extended to 4 years, the ORs decreased but remained significant in the same order. The ORs at 4 years were 3.25 (95% CI, 2.88–3.68) for GHs, 2.60 (95% CI, 2.19–3.07) for TGHs, and 1.86 (95% CI, 1.56–2.22) for hospitals.

**Figure 1:**
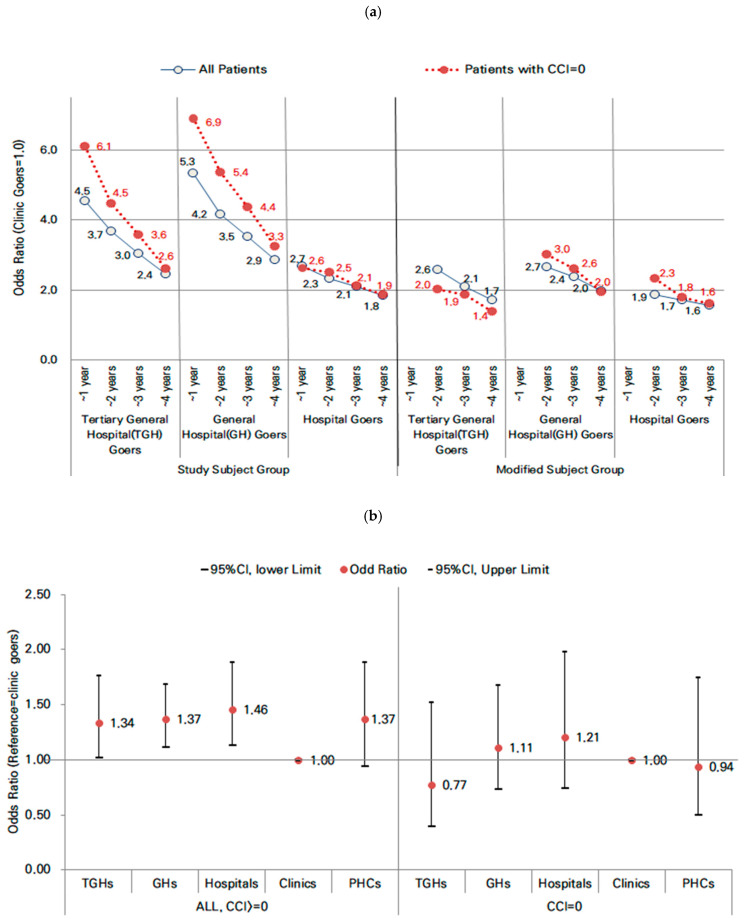
Changes in Health Outcomes by Patient Group and CCI. USC, usual sources of care (Reference = clinics; patients who frequented clinics as their USC): reference to Table 2 and Table 3. Odds Ratio of Diabetes-related Hospitalization (**a**), Odds Ratio of All-cause Death after 3 years since the diagnosis (**b**).

In the modified subject group, which excluded patients who had been hospitalized within 1 year, all ORs decreased by almost half compared to previous hospitalization. However, the ORs for hospitalization were still significantly higher for those visited hospitals, GHs, and TGHs than clinic goers. The OR for hospitalization within 2 years was 2.65 (95% CI, 2.25–3.11) for GH goers, 2.58 (95% CI, 2.11–3.16) for TGH goers, and 1.86 (95% CI, 1.49–2.34) for hospital goers, significantly higher than those for clinic goers. Considering only patients with a CCI of 0 in the modified subject group, the ORs for hospitalization in those who visited TGHs were lower for all periods compared to the GH and hospital goers (Table 2, Figure 1). Figure 1 shows the changes in ORs over the study period according to patient group and CCI level at a glance. In the modified subject group, the changes over time were similar to those of the overall cohort. In patients with a CCI of 0 in the modified subject group, the OR for TGH hospitalization decreased by almost half and was lower than those for GHs and hospitals (hospitals: OR, 1.60, 95% CI, 1.29–1.99; GHs: OR, 1.95, 95% CI, 1.65–2.30; TGHs: OR, 1.39, 95% CI, 1.08–1.78).

Regarding the risk of death, compared to patients who frequented clinics, the ORs of death in 4 years were highest among patients who frequented hospitals (OR, 1.46; 95% CI, 1.13–1.89), GHs (OR, 1.37; 95% CI, 1.11–1.69), and TGHs (OR, 1.34; 95% CI, 1.02–1.77). However, the differences became statistically nonsignificant among patients with a CCI of 0 (Table 3, Figure 1).

## 4. Discussion

We traced the outcomes of patients diagnosed with type 2 diabetes over a period of 4 years following their diagnosis and examined whether there were differences in hospitalization and mortality rates depending on the type of USC used. Our analyses revealed that compared to those who frequented clinics, patients whose USCs were hospitals, GHs, or TGHs were more likely to be hospitalized for diabetes or related diseases (cardiovascular, or renal) at all time points following diagnosis. In the group of mild patients with no hospitalizations within 1 year after diagnosis and a CCI of 0, the OR for TGH hospitalizations decreased to below those for GHs and hospitals. With respect to mortality, hospital, GH, and TGH goers had significantly greater ORs for death in 4 years following their diagnosis, compared to those choosing clinics as their USC. However, the differences in the odds among institution type became statistically nonsignificant in patients with a CCI of 0.

These results provide additional evidence that, for patients with early and mild type 2 diabetes, the value of care is better from primary care clinics. The perceived positive outcomes of the clinic goers in this study (relative to the other USCs) may be due to the receipt of more continuous care. A high number of patients far from their home visiting large hospitals, such that their capacity is exceeded, may undermine the provision of appropriate care, resulting in the deterioration of the patients’ conditions [23]. During our study period, patients who used clinics as their USC had a higher number of outpatient visits and higher MPRs than those who visited larger hospital. It has been shown previously that the higher the MPR, the lower the risk of subsequent hospital admission or mortality [22].

The Korean healthcare delivery system is divided into two levels. The first level includes clinics, hospitals, and GHs, among which patients can choose freely. Second-level services are only provided by TGHs which have specific qualifications as certified by the Ministry of Health; here, patients must present referrals issued from first-level healthcare institutions [24]. However, there are virtually no restrictions on Koreans’ access to family medicine departments or emergency care facilities with regard to TGHs [8,25]. Consequently, clinics have been competing with the outpatient services of hospitals, GHs, and even TGHs to provide primary care. An increase in patients’ preference for TGHs has led to a continuous erosion of the market share held by clinics for outpatient care. As of 2018, there were 63,388 medical institutions in Korea (not counting PHCs, midwiferies, and pharmacies), with TGHs and GHs making up only 0.5% (0.06%, [*n* = 42] and 0.46% [*n* = 311], respectively). However, of the NHI spending on outpatient services of healthcare institutions in 2018 alone, 28.3% went to TGHs and GHs (15.1% and 13.2%, respectively) [26]. A major reason for the increasing concentration of patients with mild conditions in larger hospitals is the prevailing perception that larger hospitals are better equipped with cutting-edge technologies, are staffed with highly qualified personnel, and offer high-end services [12]. However, this is merely a perception, rather than the objective reality [27], because the public have not been provided with sufficient empirical data to make informed judgments regarding the quality and value of the various types of healthcare institutions.

To reinforce a healthcare delivery system based on primary care, the Korean government has introduced diverse policy changes. In 2009, for instance, patients’ copayment rates at TGHs increased from 50% to 60%. Fifty or so mild conditions, including the common cold, hypertension, and diabetes, were also listed for care by primary-level care institutions, which means that patients visiting larger hospitals (general and above) for these listed conditions would have to pay more for drugs and supplies [28]. In addition, since 2011, the Korean government has operated a quality assessment and incentive program for clinics, aimed at promoting quality care for patients with diabetes or hypertension. The program not only provides subsidies to these ends but was also designed to increase public trust confidence in clinics as primary care providers. However, up until recently, patients’ preference for larger hospitals has continued, even for mild conditions [12,25,28]. Of the outpatient service charges covered by the NHI program from 2005 to 2018, claims from TGHs and GHs increased by 6.2 percentage points from 22.1% to 28.3%. By contrast, claims from clinics dropped by 9.9 percentage points, from 52.5% to 42.6% [28]. The shrinking representation of clinics for the treatment of patients with mild conditions has led to a decline in the role of primary care providers within the healthcare system of Korea [23].

Policy has failed to discourage Koreans from frequenting larger hospitals for mild conditions. One possible reason for this policy failure may be that Korean policymakers have been unable to leverage scientific data to convince the Korean public that clinics provide better care for diabetes and other similar diseases. The quality of diabetic care has been measured in a restricted manner, for example, based on process quality such as numbers of performing HbA1c tests from NHI claims information, and a limited bonus pay system for clinics was implemented. Furthermore, there is no quality-related incentive program for outpatient diabetic care of hospital level institutions as of 2020. The quality assessment and bonus scheme for clinics’ diabetic care is perceived as a route in which the government compensate clinics’ revenue loss due to patients’ concentration to TGHs, GHs, and hospitals.

This study has provided scientific evidence that could encourage diabetic patients to preferentially visit clinics. Especially, the 4-year follow-up could be important from the perspective of patients because studies have compared the long-term outcomes of diabetes patients according to their USC (i.e., institution type). Second, the results suggest the need for better quality-control-based policy measures for all diabetes-related medical institutions other than clinics, which have been and continue to be monitored for their quality of care in Korea. The higher risk of adverse events in GHs or hospitals than in TGHs for mild patients, as found in this study, indicates that Korea’s NHI healthcare delivery system may have covered the costs of unnecessary hospital outpatient service use and hospital admissions, at least for early and mild type 2 diabetic patients. Third, the results confirmed that outcome measures should be adjusted based on patient risk factor profiles. The differences in risk of hospitalization and mortality among different patient groups show the importance of adjusting for disease severity when comparing clinical outcomes [29]. Lastly, the findings are representative of all mild adult diabetic patients without complications diagnosed with type 2 diabetes in 2011, according to NHIC data.

Some shortfalls of this study must be acknowledged. First, we targeted relatively new diabetic patients. Thus, the differences in health outcomes for long-term patients are unknown. This study aimed to clarify the most appropriate source of care for diabetic patients with mild symptoms in the early stage of the disease, and the results cannot be generalized to all diabetes patients. Nevertheless, it is important to keep in mind that early diagnosis and intervention are crucial to the management of diabetes, and that the quality of care patients receive early on has decisive effects on their eventual health outcomes. Second, we excluded the patients who had ever been hospitalized for ischemic heart disease, stroke, renal disease, or cancer during the 12 months before their first diagnosis to define subjects with mild symptoms. This was to keep out subjects with higher risks for complications. However, there might still be some patients who were undiagnosed but severe among study subjects because we did not confirm mild diabetes with A1C3 level of fasting blood glucose level. Nevertheless, it would be less likely the case, as we had extracted patients who had no disease history of diabetes during the long period of one year in the NHI claim data covering the entire population. In the modified subject group, which excluded patients who had been hospitalized within 1 year, all ORs decreased by almost half compared to subjects before the modification in this study (Table 2). Third, we have not ascertained the exact causes for which patients frequenting clinics had lower hospitalization and mortality rates than patients frequenting larger hospitals as their USC. Although patients seeking treatment at clinics resulted in higher numbers of outpatient visits and had higher MPRs, on average, we could not conclude definitively that these two factors played a decisive role in health outcomes. Fourth, disparities in access to medical care were not examined. The results that diabetic patients living outside Seoul, earning a lower income, and receiving medical aid tended to not seek care from TGHs may be attributed to financial limitations and proximity to care locations [27]. This preference for TGHs is particularly strong among the educated or well-off [26], and the results of this study support this finding. More detailed studies controlling for it are needed in the future. Finally, disease severity and patient risk factors may have not been adequately controlled for despite our conservative approach to the analyses. Nevertheless, the results of this study could guide patients with mild diabetes toward institutions that provide higher-value care.

To improve primary care for diabetics in Korea, it is important to first improve the quality assessment and related measures over all related medical institutions in the healthcare system, as opposed to focusing only on clinics. In addition, this measurement should be linked to quality or value-based pay for performance programs opened to the public. Considering the potential of primary care, which could control social determinants of health, the development of inter-sector policies beyond the health sector should be consolidated in the direction of providing more efficient, effective, and equitable care [20]. These efforts should be undertaken based on scientifically sound outcome measures by adjusting for patient risk or disease severity [29].

## 5. Conclusions

In summary, in the early stages of diabetes, i.e., when the disease is mild, our results showed that patients could receive better care from primary care institutions, such as clinics, than from larger hospitals, but they might experience unnecessary use of hospital outpatient care.

This study indicates that clinics could be a more cost-effective option for newly diagnosed mild diabetes patients. While cost was not considered in our analyses, it has been shown that the diabetic care offered by clinics is more affordable than that provided by larger hospitals [30]. This study provides additional evidence that higher value from primary care clinics would be highly expected for early and mild type 2 diabetics. Promoting an informed, patient-centered decision toward primary care clinics would contribute to improving the value of the healthcare delivery system.

Our results suggest the necessity of enhancing long-term outcomes-based interventions to redirect chronic patients to primary care clinics. Enhancing primary care will have contributed to control unnecessary use of hospital outpatient care and avoidable hospital admissions at the same time. In the future, the further study to ascertain the exact causes for which patients’ frequent clinics had lower hospitalization and mortality than patients visiting larger hospitals as their USC would be needed.

## Figures and Tables

**Table 1:** Patient Characteristics and Outpatient Care Use by Usual Source of Care (USC) Type.

	Types of USC						*p*-Value *
	Total	TGHs	GHs	Hospital	Clinic	PHCs	
	80,540 (100)	7309 (9.1)	11,989 (14.9)	6399 (8.0)	52,885 (65.7)	1958 (2.4)	
Patients’ Characteristics							
Sex							
Male	44,129 (54.8)	4157 (56.9)	7203 (60.1)	3864 (60.4)	27,809 (52.6)	1096 (56.0)	<0.001
Female	36,411 (45.2)	3152 (43.1)	4786 (39.9)	2535 (39.6)	25,076 (47.4)	862 (44.0)	
Age, y (mean ± SD) ^†^	55.9 ± 12.4	53.2 ± 12.7	53.6 ± 12.6	54.5 ± 12.4	56.7 ± 12.2	62.3 ± 10.9	<0.001
20–44	14,768 (18.3)	1803 (24.7)	2913 (24.3)	1315 (20.6)	8618 (16.3)	119 (6.1)	<0.001
45–54	23,469 (29.1)	2187 (29.9)	3560 (29.7)	2078 (32.5)	15,287 (28.9)	357 (18.2)	
55–64	21,470 (26.7)	1900 (26.0)	3148 (26.3)	1651 (25.8)	14,170 (26.8)	601 (30.7)	
65+	20,833 (25.9)	1419 (19.4)	2368 (19.8)	1355 (21.2)	14,810 (28.0)	881 (45.0)	
Income level							
Medical Aid	4491 (5.6)	179 (2.5)	707 (5.9)	465 (7.3)	3008 (5.7)	132 (6.7)	<0.001
Decile 1–2	11,895 (14.8)	813 (11.1)	1567 (13.1)	997 (15.6)	8181 (15.5)	337 (17.2)	
Decile 3–4	11,126 (13.8)	794 (10.9)	1559 (13.0)	995 (15.6)	7490 (14.2)	288 (14.7)	
Decile 5–6	13,418 (16.7)	1043 (14.3)	1988 (16.6)	1086 (17.0)	8989 (17.0)	312 (15.9)	
Decile 7–8	17,182 (21.3)	1589 (21.7)	2696 (22.5)	1368 (21.4)	11,106 (21.0)	423 (21.6)	
Decile 9–10	22,428 (27.8)	2891 (39.6)	3472 (29.0)	1488 (23.3)	14,111 (26.7)	466 (23.8)	
Residence							
Seoul	16,976 (21.1)	2530 (34.6)	2123 (17.7)	1025 (16.0)	10,895 (20.6)	403 (20.5)	<0.001
Metropolitan	20,251 (25.1)	1483 (20.3)	3180 (26.5)	1735 (27.1)	13,568 (25.7)	285 (14.6)	
City and County	43,313 (53.8)	3296 (45.1)	6686 (55.8)	3639 (56.9)	28,422 (53.7)	1270 (64.9)	
CCI							
0	35,811 (44.5)	2974 (40.7)	5506 (45.9)	3078 (48.1)	23,151 (43.8)	1102 (56.3)	<0.001
1	20,289 (25.2)	1756 (24.0)	2804 (23.4)	1522 (23.8)	13,725 (26.0)	482 (24.6)	
2+	24,440 (30.3)	2579 (35.3)	3679 (30.7)	1799 (28.1)	16,009 (30.3)	374 (19.1)	
Outpatient Care Use (mean ± SD) ^†^							
No. Outpatient Visits	20.6 ± 17.9	12.1 ± 10.6	15.4 ± 13.1	18.0 ± 15.5	23.2 ± 19.4	20.2 ± 13.9	<0.001
MPR	0.41 ± 0.41	0.34 ± 0.40	0.41 ± 0.40	0.37 ± 0.39	0.42 ± 0.42	0.50 ± 0.41	<0.001

* Chi-square test; ^†^ ANOVA, analysis of variance. USC, usual sources of care; TGHs, tertiary general hospitals; GHs, general hospitals; PHCs, public health centers; CCI, Charlson Comorbidity Index; SD, standard deviation; MPR, medication possession ratio.

**Table 2:** Diabetes-related Hospitalization by USC Type.

Hospitalization	Types of USC				
	TGHs	GHs	Hospitals	Clinics	PHCs
Study Subject					
All (*n* = 80,540), *n* (%)	7309 (9.07)	11,989 (14.89)	6399 (7.95)	52,885 (65.66)	1958 (2.43)
In 1 year, N. of cases (%)	279 (0.35)	573 (0.71)	162 (0.20)	511 (0.63)	15 (0.02)
OR * (95% CI)	4.54 (3.90–5.28)	5.33 (4.72–6.03)	2.68 (2.24–3.21)	1.00	0.72 (0.43–1.21)
In 2 years, N. of cases (%)	409 (0.51)	808 (1.00)	256 (0.32)	952 (1.18)	37 (0.05)
OR * (95% CI)	3.68 (3.26–4.16)	4.16 (3.78–4.59)	2.32 (2.02–2.67)	1.00	0.96 (0.69–1.34)
In 3 years, N. of cases (%)	493 (0.61)	995 (1.24)	336 (0.42)	1415 (1.76)	55 (0.07)
OR * (95% CI)	3.03 (2.72–3.37)	3.53 (3.24–3.84)	2.09 (1.84–2.36)	1.00	0.95 (0.72–1.25)
In 4 years, N. of cases (%)	601 (0.75)	1211 (1.50)	450 (0.56)	2170 (2.69)	81 (0.10)
OR * (95% CI)	2.44 (2.21–2.68)	2.85 (2.64–3.10)	1.84 (0.71–1.12)	1.00	0.89 (0.71–1.12)
CCI ^†^ = 0 (*n* = 35,811), *n* (%)	2974 (8.30)	5506 (15.37)	3078 (8.60)	23,151 (64.65)	1102 (3.08)
In 1 year, N. of cases (%)	115 (0.32)	264 (0.74)	60 (0.17)	173 (0.48)	7 (0.02)
OR * (95% CI)	6.09 (4.77–7.77)	6.90 (5.67–8.40)	2.63 (1.96–3.54)	1.00	0.76 (0.36–1.63)
In 2 years, N. of cases (%)	140 (0.39)	339 (0.95)	95 (0.27)	296 (0.83)	15 (0.04)
OR * (95% CI)	4.46 (3.61–5.50)	5.36 (4.57–6.30)	2.50 (1.98–3.17)	1.00	0.92 (0.54–1.55)
In 3 years, N. of cases (%)	166 (0.46)	404 (1.13)	119 (0.33)	445 (1.24)	21 (0.06)
OR * (95% CI)	3.57 (2.96–4.30)	4.37 (3.80–5.03)	2.12 (1.73–2.61)	1.00	0.83 (0.53–1.30)
In 4 years, N. of cases (%)	190 (0.53)	470 (1.31)	163 (0.46)	710 (1.98)	37 (0.1)
OR * (95% CI)	2.60 (2.19–3.07)	3.25 (2.88–3.68)	1.86 (1.56–2.22)	1.00	0.89 (0.64–1.25)
Modified Subject: Excluding patients hospitalized within 1 year from study subject					
All (*n* = 79,000), *n* (%)	7030 (8.90)	11,416 (14.45)	6237 (7.89)	52,374 (66.30)	1943 (2.46)
In 2 years, N. of cases (%)	130 (1.85)	235 (2.06)	94 (1.51)	441 (0.84)	22 (1.13)
OR * (95% CI)	2.58 (2.11–3.16)	2.65 (2.25–3.11)	1.86 (1.49–2.34)	1.00	1.25 (0.81–1.92)
In 3 years, N. of cases (%)	214 (3.04)	422 (3.70)	174 (2.79)	904 (1.73)	40 (2.06)
OR * (95% CI)	2.08 (1.79–2.43)	2.37 (2.10–2.67)	1.72 (1.45–2.02)	1.00	1.08 (0.78–1.49)
In 4 years, N. of cases (%)	322 (4.58)	638 (5.59)	288 (4.62)	1659 (3.17)	66 (3.4)
OR * (95% CI)	1.72 (1.52–1.95)	1.97 (1.80–2.17)	1.56 (1.37–1.78)	1.00	0.95 (0.73–1.22)
CCI ^†^ = 0 (*n* = 35,192), *n* (%)	2859 (8.12)	5242 (14.90)	3018 (8.58)	22,978 (65.29)	1095 (3.11)
In 2 years, N. of cases (%)	25 (0.87)	75 (1.43)	35 (1.16)	123 (0.54)	8 (0.73)
OR * (95% CI)	2.01 (1.30–3.11)	3.00 (2.24–4.02)	2.32 (1.58–3.39)	1.00	1.08 (0.53–2.23)
In 3 years, N. of cases (%)	51 (1.78)	140 (2.67)	59 (1.95)	272 (1.18)	14 (1.28)
OR * (95% CI)	1.85 (1.36–2.52)	2.59 (2.10–3.20)	1.79 (1.35–2.38)	1.00	0.85 (0.50–1.47)
In 4 years, N. of cases (%)	75 (2.62)	206 (3.93)	103 (3.41)	537 (2.34)	30 (2.74)
OR * (95% CI)	1.39 (1.08–1.78)	1.95 (1.65–2.30)	1.60 (1.29–1.99)	1.00	0.91 (0.63–1.33)

* Adjusted for age, gender, residence, income level, and Charlson Comorbidity Index (CCI). ^†^ based on diagnosed first and secondary diseases for one year ahead of diagnosis. USC, usual sources of care; TGHs, tertiary general hospitals; GHs, general hospitals; PHCs, public health centers; OR, odds ratio; CI, confidence interval.

**Table 3:** All-cause Mortality by USC Type.

Mortality	Types of USC				
	TGHs	GHs	Hospitals	Clinics	PHCs
All patients (*n* = 80,540)					
Patients, *n* (%)	7309(9.07)	11,989(14.89)	6399 (7.95)	52,885 (65.66)	1958 (2.43)
In 4 years, N. of deaths (%)	60(0.07)	117(0.15)	71 (0.09)	437 (0.54)	30 (0.04)
OR * (95% CI)	1.34(1.02-1.77)	1.37(1.11-1.69)	1.46 (1.13-1.89)	1.00	1.37 (0.94-1.89)
Patients with CCI ^†^ of = 0 (*n* = 35,811)					
Patients, *n* (%)	2974(8.30)	5506(15.38)	3078(8.60)	23,151(64.65)	1102(3.08)
In 4 years, N. of deaths (%)	9(0.03)	27(0.08)	18(0.05)	137(0.38)	11(0.03)
OR * (95% CI)	0.77(0.39-1.53)	1.11(0.73-1.68)	1.21 (0.74-1.99)	1.00	0.94 (0.50-1.75)

* adjusted for age, gender, residence, income level, and Charlson Comorbidity Index (CCI). ^†^ based on diagnosed first and secondary diseases for one year ahead of diagnosis. USC, usual sources of care; TGHs, tertiary general hospitals; GHs, general hospitals; PHCs, public health centers; OR, odds ratio; CI, confidence interval.

## Data Availability

Not applicable.
